# A computational study of the influence of synaptic cooperativity on synaptic plasticity in a hippocampal CA1 pyramidal cell

**DOI:** 10.1186/1471-2202-13-S1-P164

**Published:** 2012-07-16

**Authors:** Bruce P Graham, Ausra Saudargiene, Stuart Cobb

**Affiliations:** 1 Computing Science and Maths, University of Stirling, Stirling, FK9 4LA, UK; 2 Informatics, Vytautas Magnus University, Kaunas, Lithuania; 3 Neuroscience and Psychology, University of Glasgow, Glasgow, UK

## 

We use a computational model to investigate the possible cooperativity between coactive synapses, both within and between input pathways, in determining plasticity outcomes at individual synapses on a hippocampal CA1 pyramidal cell (PC). The detailed compartmental model of the PC, based on a reconstructed morphology, contains distributions of core ion channels that control voltage transients and calcium entry in the soma and dendrites. Of particular note are the increasing density of A-type potassium channels (K_A_) with distance in the apical dendrites that provide strong filtering of voltage transients, and high-voltage-gated calcium channels (VGCC) in the dendrites and spines that may contribute to calcium entry in spine heads. Groups of spatially diffuse or clustered excitatory synapses on spines in dendritic layers stratum lacunosum-moleculare (SLM), stratum radiatum (SR) and stratum oriens (SO) are stimulated with single spikes or high frequency bursts. Synaptic currents are mediated by AMPA and NMDA receptors. A proportion of the NMDAR-mediated current is carried by calcium ions. As an indicator of likely synaptic plasticity, the peak calcium level (pCa) obtained in each spine head is measured.

**Figure 1:**
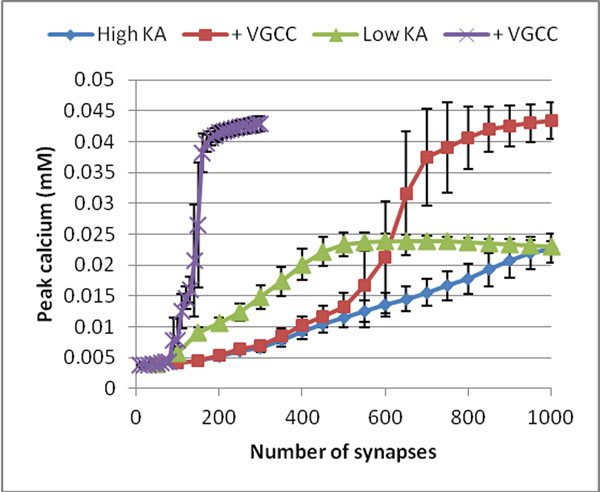
Average spine head peak calcium due to single synchronous stimulation of different numbers of synapses in SLM, in four cell configurations (1) high (standard) K_A_, (2) high K_A_ with VGCC, (3) low (one third) K_A_, (4) with VGCC. Error bars show standard deviation.

Figure 1 shows results from typical simulations. Peak calcium initially is a somewhat linear function of the number of synchronously active synapses, until saturation. The gain is higher if the density of K_A_ is reduced. With VGCCs, pCa becomes a nonlinear, sigmoidal function.

Other results show that presynaptic burst stimulation is much more effective at raising spine head calcium levels than single stimuli. Similarly, small numbers of coactive synapses on a single oblique dendrite can achieve high pCa. Activity in one layer, such as SLM can affect pCa in another layer (SR) but only if K_A_ is reduced.

In summary, burst stimulation and colocalised activity are most likely to lead to high pCa and hence LTP. Cooperativity across dendritic pathways is possible, but needs specific cellular and stimulation conditions.

